# Negative-mode mass spectrometry in the analysis of invertebrate, fungal, and protist N-glycans

**DOI:** 10.1002/mas.21693

**Published:** 2021-05-06

**Authors:** Alba Hykollari, Katharina Paschinger, Iain B. H. Wilson

**Affiliations:** 1 Department für Chemie, Universität für Bodenkultur Wien, Wien, Austria; 2 VetCore Facility for Research, Veterinärmedizinische Universität Wien, Wien, Austria

**Keywords:** MALDI-TOF MS, anionic glycans, zwitterionic glycans, invertebrates

## Abstract

The approaches for analysis of N-glycans have radically altered in the last 20 years or so. Due to increased sensitivity, mass spectrometry has become the pre-dominant method in modern glycomics. Here, we summarize recent studies showing that the improved resolution and detection by matrix-assisted laser desorption/ionization time-of-flight mass spectrometry (MALDI-TOF MS) has contributed greatly to the discovery of a large range of anionic and zwitterionic N-glycan structures across the different kingdoms of life, whereby MALDI-TOF MS in negative mode is less widely performed than in positive mode. However, its use enables the detection of key fragments indicative of certain sugar modifications such as sulfate, (methyl) phosphate, phosphoethanolamine, (methyl)ami-noethylphosphonate, glucuronic, and sialic acid, thereby enabling certain isobaric glycan variations to be distinguished. As we also discuss in this review, complementary approaches such as negative-mode electrospray ionization-MS/MS, Fourier-transform ion cyclotron resonance MS, and ion mobility MS yield, respectively, cross-linkage fragments, high accuracy masses, and isomeric information, thus adding other components to complete the jigsaw puzzle when defining unusual glycan modifications from lower organisms.

## 1 Introduction

As compared to genomics and proteomics with their often standardized workflows, glycomics still presents a significant challenge, especially with nonmammalian samples. While the bulk of glycomics is focused on mammalian species, especially either in terms of biomarkers or recombinant pharmaceuticals, the analysis of nonvertebrate N-glycans receives less attention. This is despite the evolutionary, medical, or veterinary importance of these species, including a variety of uni- and multicellular parasitic eukaryotes, model organisms, or vectors for parasitic or viral diseases. Nonmammalian glycans, for example, from parasites, are either binding partners for lectins in invertebrate intermediate hosts or ligands for proteins of the mammalian immune system; they also undoubtedly have roles in terms of self/nonself recognition in any species (Cheng et al., 2010; Martini et al., 2019; Springer & Gagneux, 2016; Vasta et al., 2007). Thus, proper definition of nonmammalian glycans enables identification of immunologically or epidemiologically relevant oligosaccharide structures and is a prerequisite for their directed synthesis. In our opinion, mere glycan profiling by mass spectrometry (MS) alone is insufficient for the analysis of glycans from an “unexplored” species, whereas even MS^*n*^ can still be ambiguous. For this reason, multiple approaches are often required, including the use of MS in both positive and negative modes as well as chemical or enzymatic treatments (Eckmair et al., 2016; Hykollari, Paschinger, et al., 2017). However, while some groups routinely use ESI-MS in negative mode, matrix-assisted laser desorption/ionization time-of-flight mass spectrometry (MALDI-TOF MS) of carbohydrates is dominated by analyses in positive mode only. A particular exception to this, especially in the case of mammalian glycomics, has been the pioneering work by David Harvey on using negative-mode MALDI-TOF-MS even for neutral glycans. His approach influenced our own work resulting in the discovery of new or unusual forms of N-glycans from invertebrate and protist species. As there are many reviews on mass spectrometry of glycoconjugates, including a recent very comprehensive one by Harvey with a focus on negative-mode MALDI-TOF-MS (Harvey, 2020), we will concentrate here on invertebrate and protist N-glycomics, complementary to a recent evolutionarily focussed overview of glycan epitopes and glycogenomic potentials in lower eukaryotes (West et al., 2020). For a listing of examples of cited studies, refer to Table 1.

## 2 Isolation of Anionic and Zwitterionic N-Glycans

A major reason for performing mass spectrometry of glycans in negative mode is to analyze those structures refractory to detection in the positive mode, especially anionic ones. One complication thereby is that neutral oligosaccharides can form negatively charged adducts, whereas anionic or zwitterionic isobaric glycans sometimes have an identical or near-identical mass as dominant neutral ones in the same biological sample. Thus, adequate preparation, fractionation, and enrichment procedures are prerequisites for detecting low-abundance “charged” glycans.

A particularly useful prefractionation step is to use graphitized carbon, which will result in neutral and anionic enriched fractions respectively when eluting with 40% acetonitrile or 40% acetonitrile supplemented with trifluoracetic acid (Chu et al., 2009; Packer et al., 1998; Paschinger & Wilson 2016). Thereby, the bulk of neutral oligomannosidic glycans can be separated from most of the sialylated, sulfated, phosphorylated, and glucuronylated structures. We have successfully performed this solid-phase extraction step on protist, mollusc, nematode, insect, and echinoderm samples as well as standard glycoproteins of mammalian origin prior to labeling the reducing terminus with 2-aminopyridine as fluorophore (Hykollari, Paschinger, et al., 2017).

Subsequently, further LC fractionation really aids the analysis—separating isomeric and isobaric structures or structures just differing in their charge status. Typically, on reversed-phase (RP) columns, most anionic glycans elute earlier, whereby sialylation or glucuronylation result in a smaller forward shift than sulfation or phosphorylation; core α1,6-fucosylation counteracts this effect to some extent (Eckmair et al., 2016, 2020). Furthermore, the well-established retention time shifts for RP-high-performance liquid chromatography (HPLC) due to the antennal extensions (3- or 6-arm) aid in deciphering structural isomers (Tomiya et al., 1988). On normal phase (NP) or mixed hydrophilic interaction/anionic exchange (HIAX) columns, not only is there a tendency for larger glycans to elute later (but with exceptions for some modifications), longer retention times are also associated with a serial increase in the number of anionic residues (Hykollari et al., 2014; Vanbeselaere et al., 2020); thereby, for example, glycans with two anionic moieties elute later than those with one or none. In our approach, such chromatographic steps are performed off-line prior to MALDI-TOF-MS; albeit not “high-throughput,” this enables the individual fractions to be subject to a range of further analyses, such as chemical or enzymatic treatments, as well as “thinking” time to “reshoot” after manual assignment. Another advantage of fractionation prior to any form of MS, including on-line fractionation, is that low-abundance structures are not suppressed—whether just by being overlooked due to the relative intensity, “in-source” loss of labile moieties, or actually due to poor ionization.

## 3 Negative-Mode MALDI-TOF Ms In Glycomics

The vast majority of MALDI-TOF MS analyses of glycans are performed in positive mode, most often with 2,5-dihydroxybenzoic acid (DHB) as a matrix as the focus is on neutral glycans; however, some fluorescent labels for glycans, such as anthranilic acid (2-aminobenzoic acid) enable per se the detection of all glycans in negative mode (Pabst et al., 2009). Examples in which negative-mode MS with DHB has been employed, include the use of intermediate pressure MALDI-FTICR-MS of glycans tagged with aminobenzoic acid, resulting in decreased “in-source” loss of sialic acid and concomitant quantification of both neutral and sialylated glycans (Reiding et al., 2017).

However, there are alternative matrices for glycomics; one is 4-chloro-α-cyanocinnamic acid, which has been used for negative-mode MALDI-TOF MS of sialylated glycans (Selman et al., 2012); another is norharmane (Nonami et al., 1998). The standard proteomics matrix, α-cyano-4-hydroxy-cinnamic acid, was used for negative-mode MALDI-TOF MS of an intact sulfated glycopeptide chain of a woodlouse hormone (Martin et al., 1999).

**Figure 1:**
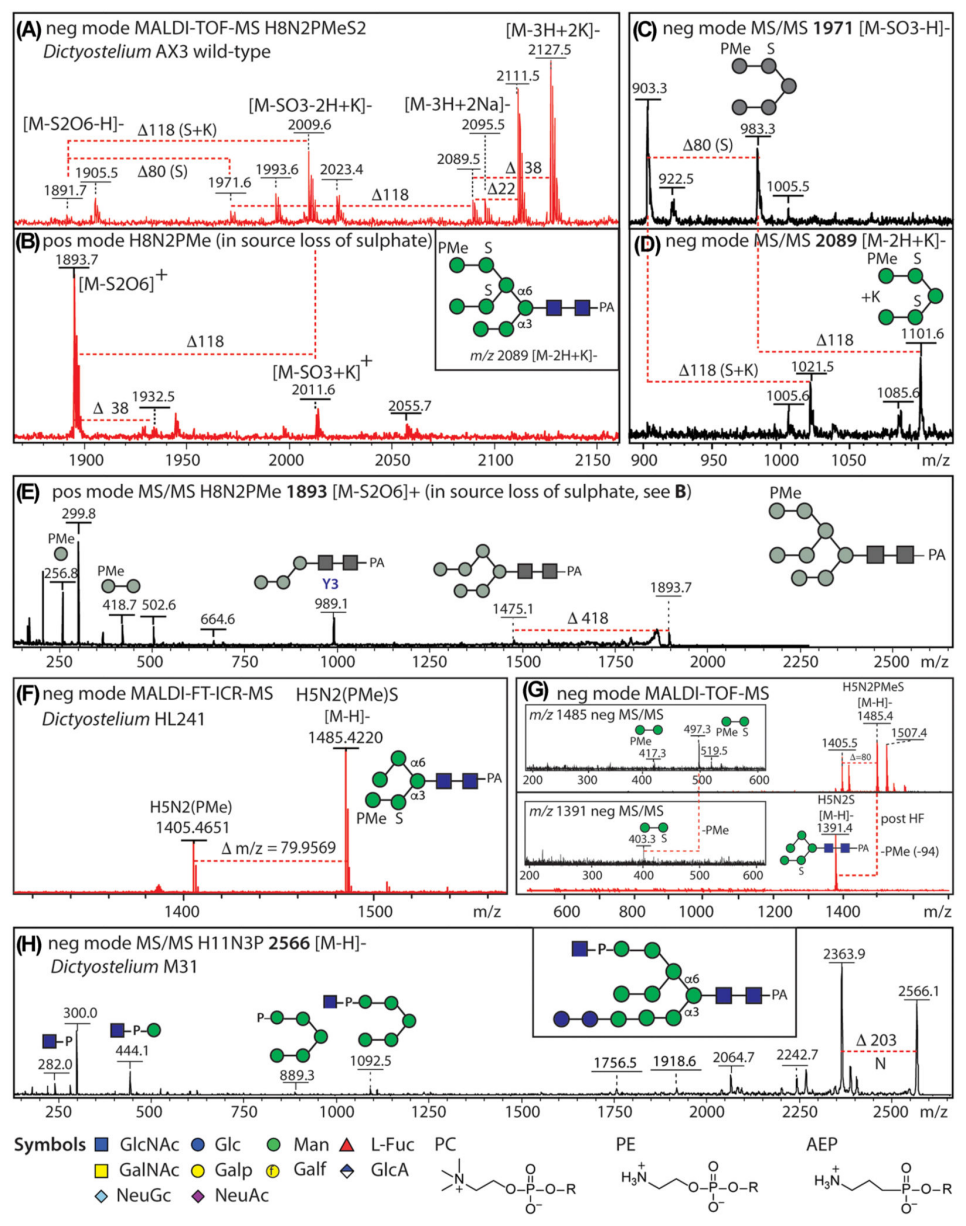
MALDI-TOF MS analysis of sulfated and phosphorylated Dictyostelium N-glycans. (A, B) Sodium and potassium adducts and in-source loss of sulfate complicate the analysis of multianionic glycans as shown for a Man_8_GlcNAc_2_S_2_PMe glycan. (C–E) Positive- and negative-mode MS/MS (laser-induced dissociation) demonstrate, respectively, the presence of methylphosphate on a Man_8_GlcNAc_2_ (Man8A isomer). (F) High-resolution negative-mode FT-ICR MALDI-TOF MS of a disubstituted N-glycan and (G) the corresponding negative-mode MALDI-TOF MS before and after HF treatment, showing the sensitivity of the phosphodiester and resistance of sulfate to this treatment. (H) Negative-mode MS/MS demonstrates the presence of a GlcNAc-1-phosphate diester on Glc_2_Man_9_GlcNAc_2_. Data are taken from the studies of Hykollari et al. (2013) and Hykollari, Malzl, et al. (2017). The N-glycans are depicted according to the Symbolic Nomenclature for Glycans with the α1,3-linked antennae lowermost (see “symbols” in the lowest panel as used throughout the article); glycan annotations in gray are for those ions resulting from “in-source” loss of sulfate. Mass differences Δ of 22, 38, 80, 94, 102, 118, and 203 correspond to Na^+^, K^+^, sulfate, methylphosphate, sodiated sulfate, potassiated sulfate, and *N*-acetylhexosamine. H, hexose; HF, hydrofluoric acid; N, *N*-acetylhexosamine; PA, pyridylamino; PMe, methylphosphate; S, sulfate [Color figure can be viewed at wileyonlinelibrary.com]

Our own approach in glycomics is to use 6-aza-2-thiothymine (ATT) as matrix as also reported by others (Juhasz & Costello, 1992; Papac et al., 1996; Wuhrer & Deelder, 2005). Generally, we use ATT without the addition of sodium, in both positive and negative modes, using the same spot, followed by MS/MS with laser-induced dissociation. Respectively, the [M+H]^+^ and [M−H]^−^ ions are the major observed forms for pyridylaminated glycans, but [M+Na]^+^ ions generally dominate when analyzing free glycans in positive mode. In negative mode, sialylated, sulfated, phosphorylated, and glucuronylated N-glycans, as well as glycosaminoglycan chains containing hexuronic acids, can be detected using ATT as matrix as well as those glycans with zwitterionic modifications such as phosphoethanolamine (PE) and aminoethylphosphonate (Eckmair et al., 2016, 2020; Hykollari et al., 2013, 2018; Vanbeselaere et al., 2018, 2020). On the contrary, many glycans with such modifications fragment better in positive mode; in the case of sulfated glycans, which show a special tendency to in-source loss of the anionic moiety in positive mode, MS/MS of [M−SO_3_]^+^ pseudomolecular ions yields important clues as to the overall glycan “backbone” (see below; Figure 1). While the MALDI-TOF-MS/MS fragmentation patterns we observe generally result from glycosidic bond cleavage in both positive and negative modes, more complicated spectra with many cross-ring cleavages are observed for ESI-MS^*n*^. The exotic nature of many nonvertebrate glycans, however, makes it difficult to unambiguously annotate all fragments; thus, we focus on searching for species variations in glycomotifs.

A further challenge is when there are multiple anionic substitutions, such as di-, tri- or tetrasulfation—in this case, even in negative mode, there is “in-source” serial loss of sulfate, but also a tendency to form multiple Na^+^ and K^+^ adducts (Figure 1A,B). To determine the actual degree of sulfation, we have found it useful to exploit the serially higher retention times on NP- or HIAX-columns for increasing numbers of sulfate residues on the same backbone (also using two-dimensional [2D] HPLC as required) as well as adding sodium salts to stabilize the sulfates prior to negative-mode MS (Hykollari et al., 2013; Kurz et al., 2013; Vanbeselaere et al., 2020).

## 4 Enzymatic and Chemical Treatments of Glycans

Very often retention time and fragmentation patterns alone will not “solve” a glycan structure—therefore, orthogonal approaches are required. For many glycosidic linkages or some anionic/zwitterionic modifications, there are enzymes or other reagents which are used for structural elucidation. For example, sialidases and glucuronidases can be used to remove sialic acid or glucuronic acid residues, but both types of enzymes can be linkage-specific (Martini et al., 2019; Eckmair et al., 2020). We have found that the typical shrimp alkaline phosphatase can remove unsubstituted phosphate (Hykollari, Malzl, et al., 2017), whereas currently commercially available sulfatases either cannot remove sulfate from glycans or are so impure that glycan signals are obscured in subsequent MS experiments.

In terms of chemical treatments, 48% aqueous hydrofluoric acid (HF) can conveniently remove phosphates, phosphodiesters (including phosphorylcholine [PC] and PE), and phosphonates as well as certain monosaccharides, including galactofuranose or α1,3-linked fucose residues (Paschinger & Wilson 2016). Recently, we observed that HF will even lactonise α2,3-linked sialic acid resulting in a loss of 18 Da (Eckmair et al., 2020), similar to the effect of 3-(3-dimethylaminopropyl)-carbodiimide/1-hydroxybenzotriazole treatment (Reiding et al., 2014), whereas sulfate is unaffected (Figure 1G). For desulfation, solvolysis with 50 mM metha-nolic hydrochloric acid (HCl) is used, but is often incomplete and is accompanied by degradation of the glycan. Nevertheless, we have successfully removed sulfate using this approach for more-abundant glycans prior to subsequent glycosidase treatments, co-elution experiments or perdeuteromethylation (Kurz et al., 2015; Vanbeselaere et al., 2020).

## 5 Negative-Mode MALDI-TOF Ms of Neutral N-Glycans

**Figure 2:**
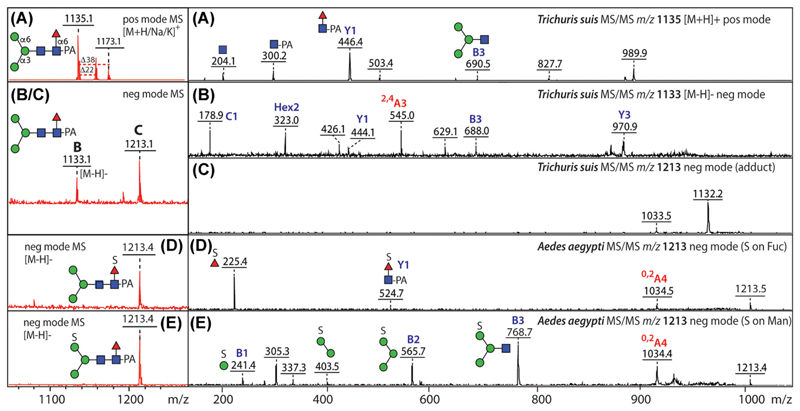
MALDI-TOF MS analysis of neutral and sulfated α1,6-fucosylated paucimannosidic glycans. (A–C) Positive- and negative-mode MS and MS/MS of an HPLC-purified neutral Man_3_GlcNAc_2_Fuc_1_ glycan from *Trichuris suis* and its negative-mode adduct. (D, E) Negative-mode MS and MS/MS of HPLC-purified Man_3_GlcNAc_2_Fuc_1_S_1_ glycans from insects with sulfation of either core fucose or mannose. Data are taken from the studies by Wilson & Paschinger (2016) and Kurz et al. (2015); structures are corroborated by mannosidase and fucosidase treatments. In all cases, the positive-mode spectra show a major [M+H]^+^ ion (A) or [M−SO_3_]^+^ ion (D, E; not shown) at *m*/*z* 1135, whereas *m*/*z* 1,213 ions are observed in negative mode. However, while the *m*/*z* 1133 [M−H]^−^ ion (B) is a molecular ion yielding various fragments including the ^2,4^A_3_ ion (shown in red) resulting from cross-ring cleavage of the second core GlcNAc residue, MS/MS of the *m*/*z* 1213 ion (C) results primarily in loss of the adduct. In contrast, MS/MS of the *m*/*z* 1213 [M−H]^−^ ions of sulfated glycans from insects (D, E) yields a number of fragments defining different locations of the sulfate moiety (on either fucose or mannose) and also a cross-ring ^0,2^A_4_ cleavage of the pyridylaminated reducing-terminal GlcNAc. [Color figure can be viewed at wileyonlinelibrary.com]

Many typical neutral glycans do not ionize so well in negative mode. However, neutral N-glycans isolated from *Schistosoma mansoni* and labeled with 2-aminobenzamide have been detected in negative mode when using ATT as matrix; MS/MS yielded cross-ring fragments comparable with those from negative-mode ESI-MS (Wuhrer & Deelder, 2005). In the case of another trematode parasite, *Fasciola hepatica*, aminobenzoic acid-labeled glycans were analyzed by negative-mode MALDI-TOF MS, including neutral paucimannosidic, oligomannosidic, and hybrid structures (Garcia-Campos et al., 2016; Ravida et al., 2016). Our own observations with pyridylaminated glycans suggest that highly abundant pauci- and oligomannosidic forms tend to be associated with adducts in negative mode and that MS/MS of their deprotonated molecular ions will yield cross-ring fragments such as the ^2,4^A cleavage of the second core GlcNAc residue (Figure 2A–C). Examples in which intact small glycoproteins have been analyzed by negative-mode MALDI-TOF MS are studies on GPI-linked glycoproteins from different *Trypanosoma* spp. carrying oligomannosidic structures (Macrae et al., 2005; Manthri et al., 2008). However, in general, as high-abundance neutral structures are not challenging to analyze, the informational gain is not great.

## 6 Negative-Mode MALDI-TOF Ms of Sulfated and Phosphorylated N-Glycans

The major advantage in using MALDI-TOF MS in the negative mode is to detect anionic glycans as deprotonated molecular ions. Hereby, many invertebrate species have been shown to have a variety of anionic modifications—sulfation being very widespread, glucuronylation, and phosphorylation less so, whereby these moieties are also present on various mammalian glycans. As both sulfate and phosphate result in an additional 80 Da on a glycan, it is important to distinguish these adequately, whereby sulfation is more difficult to deal with, as it is more labile in a mass spectrometer. Furthermore, when using permethylation, sulfated glycans are often overseen as these are in the aqueous phase upon organic extraction and so special purification steps are required (Kumagai et al., 2013; Yu et al., 2009). As mentioned above (Section 4), not only do sulfate and phosphate have contrasting sensitivity to chemical and enzymatic treatments but also ionize differently in MALDI-TOF MS. On the basis of these properties, we can be confident when proposing whether an N-glycan is sulfated or phosphorylated when using a MALDI-TOF instrument with a standard resolution.

In protists, both sulfation and phosphorylation of glycans occur in *Dictyostelium discoideum* (social amoeba or slime mold); both older work (Freeze & Wolgast, 1986; Gabel et al., 1984) and our own MALDI-TOF-MS-based studies, backed up by high-resolution negative-mode FT-MS, showed the presence of these moieties (Hykollari et al., 2013). In this organism, the phosphate (generally methylated) is on terminal mannose residues and the sulfate often on subterminal ones as indicated by the corresponding fragments: *m*/*z* 255/417 and *m*/*z* 257/419 for Hex_1–2_PMe in both MS/MS modes and *m*/*z* 403 fragments for Hex_2_S_1_ in negative mode, whereby glycans or fragments with two or more anionic moieties were detected as, for example, [M−2H+Na/K]^−^ ions (Figure 1A–G). Overall, other than using an ultrahigh resolution FT-MS instrument, phosphate and sulfate can be distinguished on a standard MALDI-TOF MS due to the different ionization properties and contrasting HF sensitivity of these two nonsugar 80 Da modifications (Figure 1F,G). In a glucosidase-mutant strain, we could also detect a phosphatase-resistant GlcNAc-1-P phosphodiester biosynthetic intermediate (Hykollari, Malzl, et al., 2017) as judged by detection of *m*/*z* 282 and 444 Hex_0-1_HexNAc_1_P B-fragments (Figure 1H).

In terms of other protists, we also observed phosphorylated N-glycans in one strain of *Trichomonas vaginalis* (Paschinger et al., 2012), whereas sulfation was reported by others as a modification of *Trypanosoma cruzi* N-glycans, but no MS/MS was shown (Barboza et al., 2005). Negative-mode MALDI-TOF-MS was also used to detect Xyl-P-modified N-glycans of the pathogenic fungus *Cryptococcus neoformans* (Park et al., 2012).

In insects, we have reported sulfation of core fucose and of mannose (Hykollari et al., 2018, 2019; Kurz et al., 2015; Stanton et al., 2017), which can be told apart by the presence of either *m*/*z* 225 or 241 negative-mode MS/MS fragments (Figure 2D,E). Others have also found sulfation of mannose residues in arthropods, as judged in part by negative-mode MALDI-TOF MS either at the level of a whole glycoprotein (Martin et al., 1999) or of released glycans (Cabrera et al., 2015, 2017); also, 6-sulfation of mannose in a lobster N-glycan was reported by NMR (van Kuik et al., 1987). In the trematode *Fasciola hepatica*, the definition of the 80-Da modification of some hybrid glycans as phosphate relied on negative-mode MALDI-FT-ICR (Fourier-transform ion cyclotron resonance) MS (Ravida et al., 2016).

**Figure 3:**
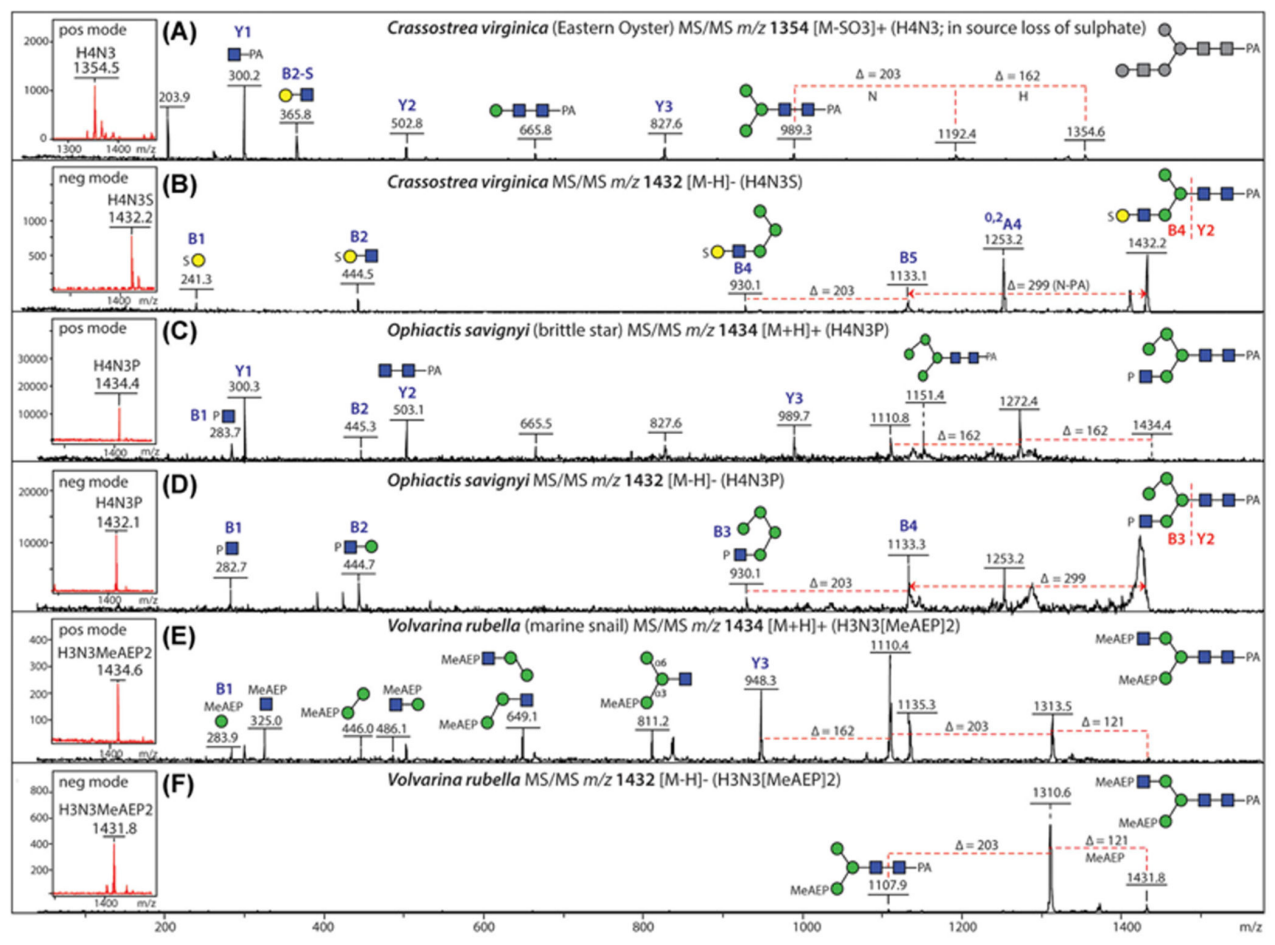
MALDI-TOF MS analysis of isobaric N-glycans. (A, B) Positive- and negative-mode MS/MS of a sulfated N-glycan (1433 Da; *m*/*z* 1354 as [M−SO_3_]^+^ or 1432 as [M−H]^−^) from the Eastern oyster showing its backbone structure and the modification of the terminal galactose. (C, D) Positive- and negative-mode MS/MS of a phosphorylated N-glycan (1433 Da; [M+H]^+^ and [M−H]^−^) from the brittle star showing the phosphorylated GlcNAc B-ions in both modes. (E, F) Positive- and negative-mode MS/MS of a methylaminoethylphosphonate (MeAEP)-modified N-glycan (1433 Da; [M+H]^+^ and [M−H]^−^) from a marine snail showing respectively B-ions derived from the MeAEP-modified GlcNAc and Man residues or the loss of MeAEP (121 Da) from the parent ion. Selected A, B, and Y ions are annotated; the depiction of antennae (α1,3-linked antennae lowermost) is based on retention time and/or glycosidase digestion data. Data are taken from Kurz et al. (2013) and Eckmair et al. (2016, 2020). [Color figure can be viewed at wileyonlinelibrary.com]

**Figure 4:**
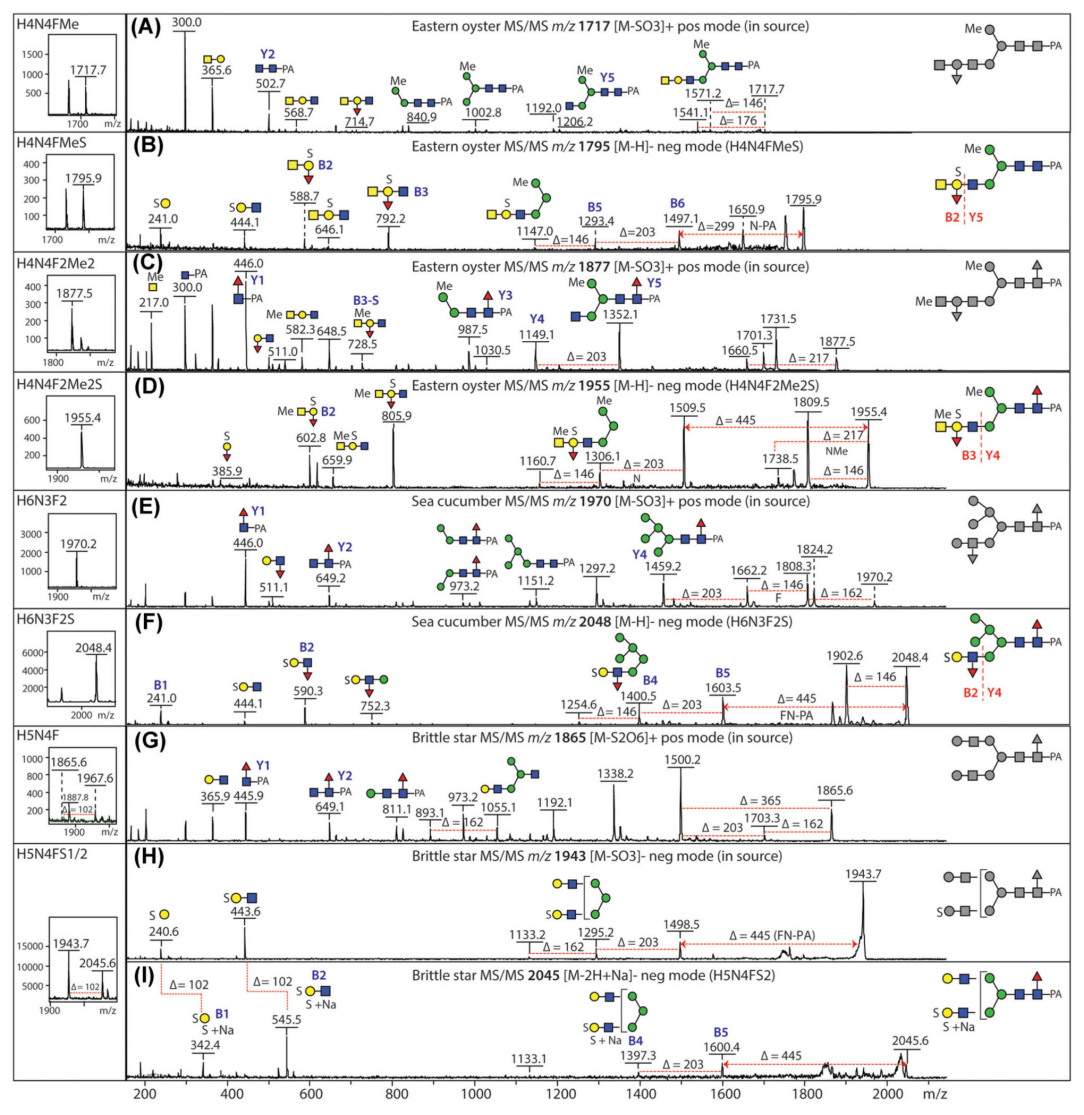
MALDI-TOF MS analysis of sulfated glycans from marine organisms. (A-D) Positive- and negative-mode MS/MS of sulfated blood group A-containing N-glycans from the Eastern oyster (*Crassostrea virginica*) also substituted with a methyl group either on the α1,6-mannose or the α1,3-linked GalNAc; MS/MS of the [M−SO_3_]^+^ pseudomolecular ions resulting from in-source loss of sulfate shows the backbone structures (in gray), whereas negative-mode deprotonated B-ions show the linkage of sulfate to the galactose of the blood group moiety. (E, F) Positive- and negative-mode MS/MS of sulfated Lewis A-containing N-glycan from a sea cucumber (*Holothuria atra*); MS/MS of the [M−SO_3_]^+^ pseudomolecular ion shows the backbone structure, whereas in the negative-mode deprotonated B ions at *m*/*z* 241 indicate sulfation of the galactose of the Lewis moiety. (G–I) Analysis of a sulfated N-glycan from a brittle star (*Ophiactis savignyi*) with MS/MS of the [M−S_2_O_6_]^+^ and [M−SO_3_]^−^ pseudomolecular ions resulting from “in-source” fragmentation and of the “intact” [M−2H+Na]^−^ molecular ion; the negative-mode *m*/*z* 342 sodiated B-ion shows disulfation of the same galactose residue and the positive-mode MS/MS aids elucidation of the backbone structure. Data are taken from Kurz et al. (2013), Vanbeselaere et al. (2020) and Eckmair et al. (2020). Selected B and Y ions are annotated. F, fucose (Δ*m/z* 146); H, hexose (Δ*m/z* 162); Me, methyl (MeHex, Δ*m/z* 176; MeHexNAc, Δ*m/z* 217); N, *N*-acetylhexosamine (Δ*m/z* 203); PA, pyridylamino; S, sulfate [Color figure can be viewed at wileyonlinelibrary.com]

Sulfate is especially abundant on N-glycans in marine species; as judged by studies on molluscs (which include the gastropods and bivalves) and echinoderms (which evolved before the vertebrates, but are deuterostomes like us), different sulfation patterns occur in a species-dependent manner. While in a marine gastropod, sulfation of mannose and GlcNAc was detected (Eckmair et al., 2016), “marine” sulfation is often located on Galβ1,3GlcNAc antennae, which correlates with an *m*/*z* 444 negative-mode B-ion, for example, as found for the Eastern oyster (a bivalve; Figure 3A,B) and the brittle star (an echinoderm). These “neo-lacto” antennae can be further modified; for instance, in the Eastern oyster, sulfation occurred partly in the context of blood group A modifications (Kurz et al., 2013) resulting in *m*/*z* 792 or 806 B-ions (Figure 4A–D); in a sea cucumber, sulfation of Lewis A (Vanbeselaere et al., 2020) was associated with *m*/*z* 590 fragments in negative mode (Figure 4E,F). It was a particular challenge to detect as many as four sulfates on selected sea cucumber glycans with substitution of either Gal, GlcNAc, and Man residues, but fractionation of glycans by HIAX-HPLC and supplementing the MALDI matrix with sodium acetate aided the detection of such structures (see also Section 3 above). In the case of a brittle star, a lower degree of sulfation was observed on glycans purified by 2D-HPLC (RP-amide followed by HIAX), but the modified units were Gal, GlcNAc, and core Fuc (Eckmair et al., 2020), whereby even a disulfated galactose was found as judged by an *m*/*z* 342 B-ion (Figure 4G–I). In addition, phosphorylation of terminal Glc or GlcNAc residues were found in respectively the sea cucumber and the brittle star (Eckmair et al., 2020; Vanbeselaere et al., 2020); in the latter case, *m*/*z* 282 and 444 fragments were observed in negative mode (Figure 3C,D).

## 7 Negative-Mode MALDI-TOF Ms of Glucuronylated and Sialylated N-Glycans

The approximate “native” mass difference for glucuronylation is 176 Da, which is isobaric with the presence of methylated hexose. However, mono-glucuronylated glycans can be easily detected in both positive and negative modes; more heavily modified larger glycans may not be so visible in positive mode, but are obvious in negative mode, although some MALDI-TOF instruments generate more in-source fragmentation (Kurz et al., 2013). As regards MS/MS, fragments corresponding to glucuronylated motifs of more-abundant glycans can be seen in negative mode, but the best MS/MS data are obtained in positive mode, even when fragmenting a corresponding “invisible” positive parent ion. The glucuronidases commercially available vary in purity and linkage specificity but are useful to prove the occurrence of glucuronic acid rather than methylated hexose.

**Figure 5:**
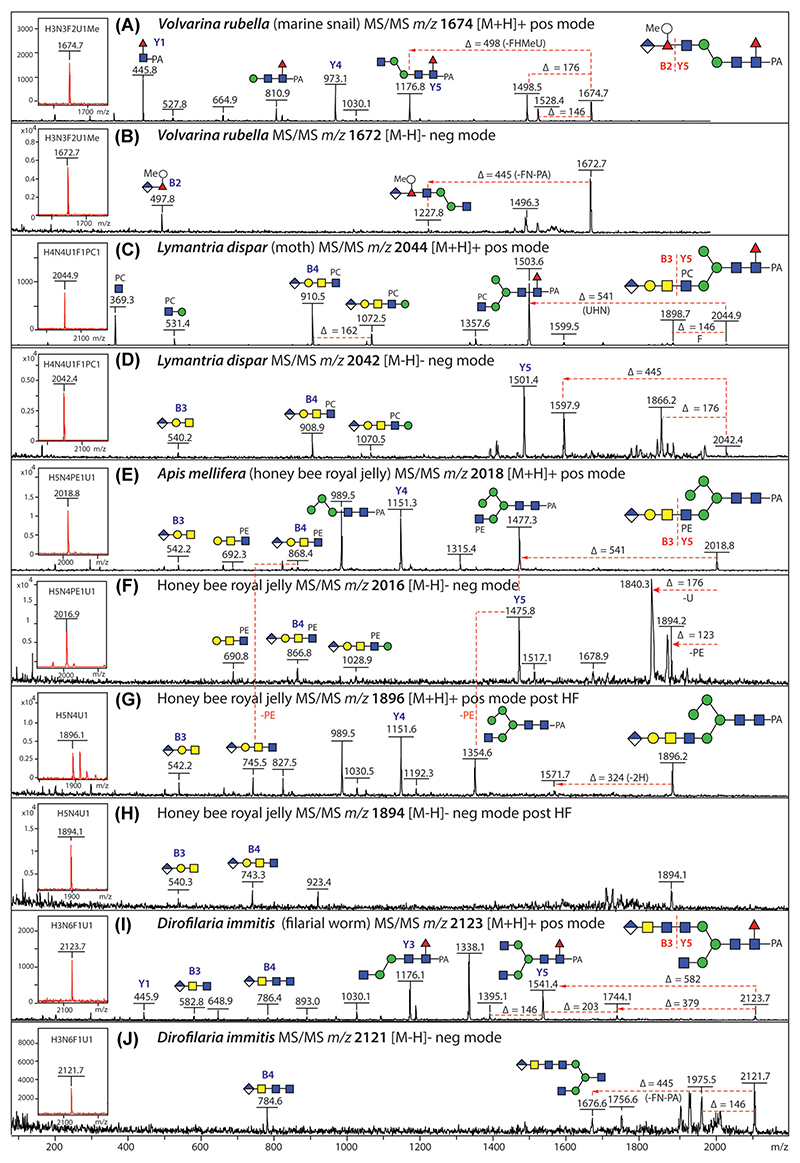
MALDI-TOF MS/MS of glucuronylated N-glycans. (A, B) Positive- and negative-mode MS/MS of an N-glycan from a marine snail carrying a branched GlcA_1_MeHex_1_Fuc_1_ motif, showing either neutral loss of this modification or the relevant negative-mode B-ion at *m/z* 497. (C, D) Positive- and negative-mode MS/MS of an N-glycan from the gypsy moth carrying a phosphorylcholine (PC)-modified glucuronylated antenna shown especially by the B-ions at *m/z* 910 and 908. (E–H) Positive- and negative-mode MS/MS before and after HF treatment of an N-glycan from royal jelly carrying a phosphoethanolamine (PE)-modified glucuronylated antenna shown especially by the B-ions at *m/z* 868 and 866, which shift to ones at *m/z* 745 and 743 after removal of PE. (I, J) Positive- and negative-mode MS/MS of an N-glycan from the canine heartworm carrying an extended glucuronylated antenna shown especially by the B-ions at 786 and 784. Data are taken from Eckmair et al. (2016); Stanton et al. (2017); Hykollari et al. (2018) and Martini et al. (2019) and were corroborated by hydrofluoric acid and glucuronidase treatments. Selected B and Y ions are annotated. F, fucose (Δ*m/z* 146); H, hexose (Δ*m/z* 162); HF, hydrofluoric acid; N, *N*-acetylhexosamine (Δ*m/z* 203); PA, pyridylamino; U, glucuronic acid (Δ*m/z* 176) [Color figure can be viewed at wileyonlinelibrary.com]

In our various studies, glucuronylation of invertebrate N-glycans is a recurrent theme and occurs in different linkages, we have found it in a marine snail linked to Fuc (Eckmair et al., 2016), in all insect species we have analyzed to date linked to Gal (Hykollari et al., 2018, 2019; Kurz et al., 2015; Stanton et al., 2017) and in a filarial nematode parasite linked to GalNAc (Martini et al., 2019). Depending on the motif and length of the antenna, negative and positive B-ions such as *m*/*z* 497/499, 540/542 and 743/745, or 581/583 and 784/786 are observed for GlcA_1_Fuc_1_HexMe_1_, GlcA_1_Gal_1_HexNAc_1-2_, and GlcA_1_HexNAc_2-3_; substitution of such antennae by a PE or PC residue results in further shifts of 123 and 165 Da (Figure 5). Complementary negative-mode LC-ESI-MS^*n*^ has also aided the definition of the relevant linkages. It is to be noted that glucuronylation is not highly abundant in terms of percentage, but using graphitized carbon, most glucuronylated glycans are in the “anionic-enriched” fraction and so their detection is enhanced when they are separated from the more-abundant oligomannosidic structures.

**Figure 6:**
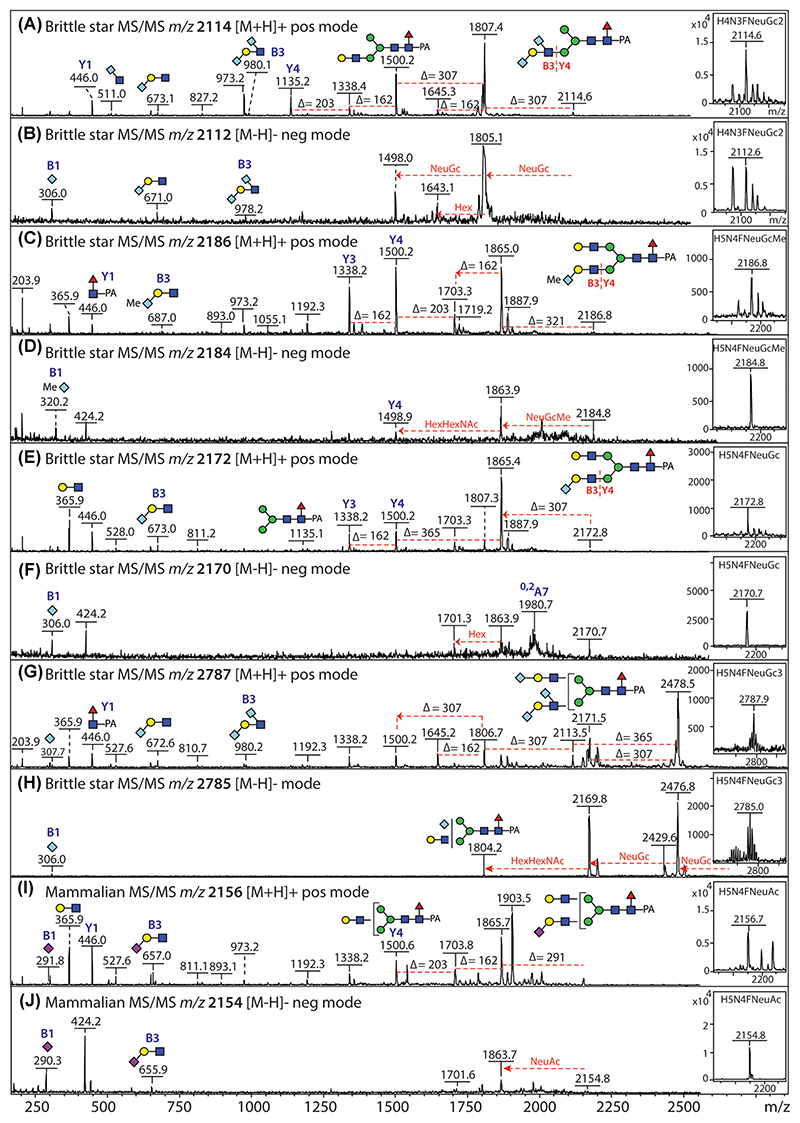
MALDI-TOF MS/MS of sialylated N-glycans. Positive- and negative-mode MS/MS of brittle star N-glycans modified with one or more *N*-glycolylneuraminic acid and methylated *N*-glycolylneuraminic acid residues (A–H; Δ*m/z* 307 and 321 Da) and a mammalian N-glycan modified with one *N*-acetylneuraminic acid residue (I, J; Δ*m/z* 291 Da). Data are taken from Eckmair et al. (2020) and were verified by sialidase or acid treatments. Selected B and Y ions are annotated; key *m/z* differences are annotated. [Color figure can be viewed at wileyonlinelibrary.com]

While sialic acid is certainly a common component of vertebrate glycans, the matter of sialylation in invertebrates is more controversial, partly due to misleading lectin blotting data. Sialic acid is also a “tricky” modification, which is labile under acidic conditions, thus hampering its detection; however, avoiding low pH where possible and by employing 2D-HPLC fractionation (RP-amide at pH 6 followed by HIAX), negative-mode MALDI-TOF-MS of brittle star N-glycans combined with positive-mode MS/MS and various treatments enabled us to determine the occurrence of *N*-glycolylneuraminic acid attached either α2,3-linked to terminal Gal or α2,6-linked to subterminal GlcNAc residues (Eckmair et al., 2020). While in the sea cucumber, the NeuGc was not methylated (Vanbeselaere et al., 2020), the NeuGc in the brittle star was frequently methylated, but the “terminal” sialic acid could nevertheless be removed with α2,3-specific sialidase S which resulted in loss of the negative-mode signal unless the glycan was also modified with a further anionic moiety such as sulfate (Eckmair et al., 2020); other sialidases will remove α2,6-sialic acid, but not that linked to the GlcNAc. Typical MS/MS B-ion fragments for sialylated N-glycans in negative and positive modes are at *m*/*z* 655/657, 671/673, 685/687, and 978/980 for NeuAc, NeuGc, MeNeuGc or two NeuGc residues attached to Gal_1_GlcNAc_1_ (Figure 6).

## 8 Negative-Mode MALDI-TOF Ms of Zwitterionic N-Glycans

Zwitterions can carry both a positive and a negative charge; whereas some bacteria have zwitterionic oligosaccharides with alternating cationic and anionic monosaccharides (Zhang et al., 2019), other glycoconjugates found in various prokaryotic and eukaryotic species naturally contain a non-sugar moiety with both an amine and phosphodiester or phosphonate (with a direct C–P bond) “bridge” to the sugar (Young et al., 2013). Examples are PE (2-aminoethylphosphate), PC (*N,N,N*-trimethyl-2-aminoethylphosphate), 2-aminoethylphosphonate (AEP), and *N*-methyl-2-aminoethylphosphonate (MeAEP; see also legend for Figure 1). Both PE and PC are found as the head groups of phospholipids; PE is also a side chain of mammalian glycosylphosphatidylinositol anchors (Roberts et al., 1988), but is seemingly otherwise absent from vertebrate glycans. On the contrary, PC endows immunomodulatory activity to glycoconjugates to which it is attached, but is also a ligand for C-reactive protein, a component of the innate immune system (Volanakis, 2001). The biological role of the phosphonates is even less clear.

In terms of mass spectrometry, glycans modified with PE (+123 Da), AEP (+107 Da), and MeAEP (+121 Da) can be detected in both positive and negative modes, whereas the trimethylation of the amine of PE results in PC (+165 Da), which is only observed in positive mode; due to their excellent ionization, PC-containing glycans and fragments dominate any positive-mode MS or MS/MS spectrum. In protists and fungi, we could find PE attached to mannose residues of N-glycans of *Trichomonas vaginalis* strains and of *Penicillium* species, as judged by detection in both positive and negative modes and sensitivity to HF treatment (Hykollari et al., 2016; Paschinger et al., 2012). In the case of the honeybee, N-glycans with PE attached to GlcNAc residues were found (Hykollari et al., 2018, 2019), contrasting to the PC found in Lepidoptera (Stanton et al., 2017). For more abundant glycans with the PE in the context of a longer antenna, the negative-mode B-fragments can be weak, but *m*/*z* 866 B-fragments are, for instance, indicative of a GlcAβ1,3-Galβ1,3GalNAcβ1,4(PE)GlcNAc motif (Hykollari et al., 2018). Analogously, a GlcAβ1,3Galβ1,3GalNAcβ1,4(PC) GlcNAc motif on an anionic lepidopteran glycan yields due to the glucuronic acid residue, an *m*/*z* 909 fragment in negative mode (Stanton et al., 2017) (Figure 5C–F).

**Figure 7:**
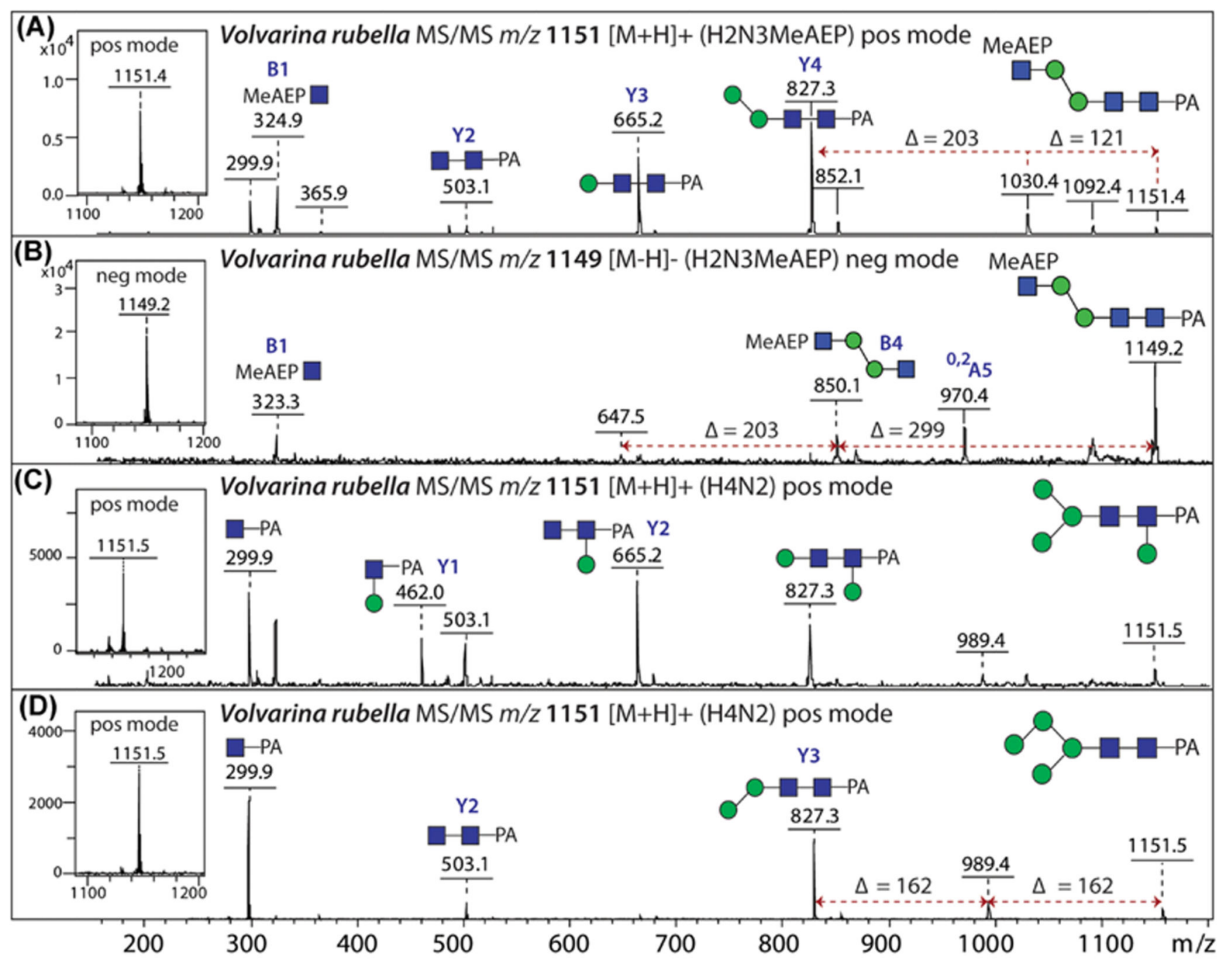
MALDI-TOF MS/MS of isobaric N-glycans from a marine snail. Positive-(A, C, D) and negative-(B) mode MS/MS of three isobaric *Volvarina rubella* N-glycans (1150 Da; i.e., either *m/z* 1151 or 1149): One structure contains methylaminoethylphosphonate (MeAEP), whereas the other two are respectively core β-mannosylated and standard isomers of Man_4_GlcNAc_2_. The glycans were pre-fractionated by RP-HPLC prior to MALDI-TOF-MS and detected as either [M+H]^+^ or [M−H]^−^ parent ions; the MeAEP and β-mannose motifs were also verified by MALDI-TOF MS/MS after respective hydrofluoric acid or β-mannosidase treatments. Selected A, B, and Y ions are annotated. Data are taken from Eckmair et al. (2016). [Color figure can be viewed at wileyonlinelibrary.com]

The routine use of negative-mode MALDI-TOF MS in our group has meant that glycans containing zwitterionic moieties could be distinguished from isobaric neutral structures. A particular example is illustrated by the N-glycomic analyses of a marine gastropod (Eckmair et al., 2016). Here, glycans of, e.g., 1150 Da could be detected in multiple HPLC fractions, but some of them yielded signals in both positive and negative modes as well as losses of 121 Da upon MS/MS (Figure 7); these properties seemed rather unusual for a Man_4_GlcNAc_2_ glycan; thus, also under consideration of the literature on mollusc glycolipids, we tried out HF treatment, which also resulted in a loss of 121 Da. Therefore, we could postulate that this glycan was not paucimannosidic, but rather carried an MeAEP attached to a GlcNAc residue (Eckmair et al., 2016). Another glycan carrying this modification is isobaric to sulfated and phosphorylated glycans from other species, but MS/MS yields different fragments (Figure 3E,F); this highlights the importance of fragmenting all glycans before annotating potential structures.

## 9 Negative-Mode Fast Atom Bombardment Mass Spectrometry (FAB-MS) of Invertebrate and Protist Glycans

Historically, FAB-MS was important in the pioneer days of glycomics; thereby, also negative-mode FAB-MS of lower eukaryotic glycans is worth briefly mentioning. For instance, methylphosphorylated N-glycans from *Dictyostelium* (Gabel et al., 1984), MeAEP-modified glycolipids from snails (Hayashi & Matsubara, 1989), sialylated glycolipids (gangliosides) from starfish (Higuchi et al., 2007), or sulfosialylated O-glycans from a sea urchin (Kitazume et al., 1996) were investigated by this approach. FAB-MS has, though, be long superseded by other MS techniques.

## 10 Negative-Mode ESI- or NSI-MS of Invertebrate, Fungal, and Protist N-Glycans

**Figure 8:**
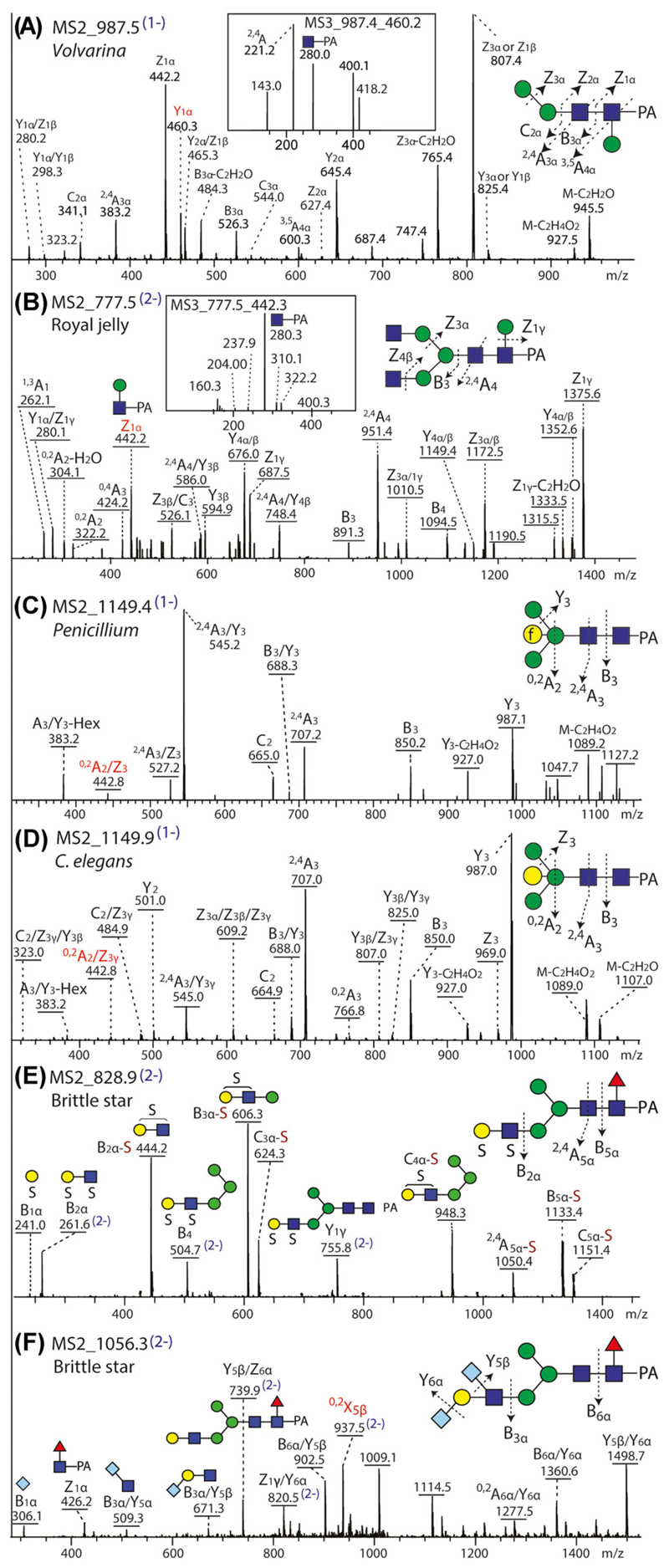
Example negative-mode ESI-MS/MS analyses of invertebrate N-glycans. (A, B) Different cross-ring cleavage fragments (MS2 and MS3) of the reducing terminal GlcNAc-PA correlate, respectively, with β1,3- or β1,6-core mannosylation of glycans from a marine snail (*Volvarina rubella*) and honeybee royal jelly. (C, D) Subtly different MS/MS spectra of isomeric glycans featuring either bisecting galactofuranose (*Penicillium verrucosum*; after jack bean α-mannosidase digestion of Hex_10_HexNAc_2_) or bisecting galactopyranose (*Caenorhabditis elegans*) with the key ^0,2^A/Z_3_ “bisected” fragment highlighted. (E, F) Sulfated B-ion fragments dominate the MS/MS of a brittle star sulfated N-glycan, whereas the sialylated B-ion fragments of a disialylated glycan from the same species are relatively weak; corresponding MALDI-TOF MS/MS of the latter are shown in Figure 6A,B. Data is taken from Eckmair et al. (2016, 2020); Hykollari et al. (2016, 2018); Yan et al. (2015); the glycans were prefractionated by RP-HPLC prior to PGC-LC-ESI-MS and were detected as either [M−H]^−^ or [M−2H]^2−^ parent ions (designated as 1- or 2-in blue) and key motifs also verified by chemical or enzymatic treatments and, in case of bisecting galactose, either GC-MS or NMR as described in the respective publications. [Color figure can be viewed at wileyonlinelibrary.com]

The typical approach of the Packer and some other laboratories is to use porous graphitised carbon inline with ESI-MS in negative mode with collision-induced dissociation for MS^*n*^ (Karlsson et al., 2004; Ashwood et al., 2019). Thereby, both neutral and anionic glycans, including isomers, can be separated and analyzed; the cross-ring cleavage collision-induced dissociation fragmentation data is frequently rich and aids definitions of linkages between residues. Examples of this method for invertebrate, protist and fungal N-glycans include analyses of: (i) *Penicillium* with bisecting galactofuranose (Hykollari et al., 2016); (ii) oligomannosidic glycans from *Euglena gracilis* (an alga) with a potential 2-aminoethylphosphonate modification (O’Neill et al., 2017); (iii) the paucimannosidic Man_3_GlcNAc_2_ glycan derived from a recombinant protein produced in *Leishmania tarentolae* (Klatt et al., 2013), (iv) sulfated and sialylated glycans from echinoderms and molluscs (Eckmair et al., 2020; Kurz et al., 2013; Vanbeselaere et al., 2020); (v) glycans from honey-bee royal jelly with β1,6-linked mannose attached to the core GlcNAc residue (Hykollari et al., 2018); (vi) various unusual modifications of glycans from a marine snail (Eckmair et al., 2016); (vii) glucuronylated glycans from a filarial nematode (Martini et al., 2019); (viii) glycans from *Caenorhabditis* with bisecting or intersecting galactose as well as galactosylated core fucose residues (Yan, Brecker, et al., 2015; Yan, Jin, et al., 2015; Yan, Vanbeselaere, et al., 2018; Yan, Wang, et al., 2018). Most of these examples are HPLC-fractionated glycans for which ESI-MS^*n*^ was used (see also Table 1 and Figure 8) in combination with our off-line MALDI-TOF-MS approach.

An alternative to LC coupling for ESI is capillary electrophoresis (Bunz et al., 2013) and negative-mode CE-MS was applied to oligomannosidic structures from a filamentous fungus, some carrying a Man-6-P phosphodiester (Sandra et al., 2004). Otherwise, samples can be infused directly into an NSI- or ESI instrument: in terms of lower eukaryotes, negative mode MS/MS without online LC has been employed to analyze permethylated sulfated N-glycans from three insect species (Kurz et al., 2015), methylated/xylosylated N-glycans from a red microalga (Levy-Ontman et al., 2011) and antennal branched fucose modifications on native N-glycans of the blue mussel (Zhou et al., 2013).

## 11 Negative-Mode FT-ICR MS of Invertebrate and Protist N-Glycans

The highest accuracy mass determination is possible with FT-ICR-MS which can easily distinguish “near-isobaric” modifications such as sulfate and phosphate (Figure 1F). For lower eukaryotes, it has been applied using a MALDI source in negative mode to N-glycans from *Dictyostelium discoideum* (slime mold) and *Fasciola hepatica* (liver fluke) yielding independent proof for mannose-bound sulfate and methylphosphate in the former (Hykollari et al., 2013) and GlcNAc-bound phosphate in the latter (Ravida et al., 2016). Recent advances in magnet technology may make MR-MS instruments more widely accessible; coupled to MALDI imaging, there is untapped potential to examine the spatial distribution of glycan types by this method in nonmammalian systems.

## 12 Negative-Mode IM-MS of Invertebrate Glycans

Although ion mobility MS was first developed some decades ago, it can be considered as an “emerging” method for glycomics. Depending on the mode of operation, it can be used to generate “clean” glycan spectra, distinguish isomeric glycans, or separate fragment ions; it can be performed coupled to either a MALDI or ESI source (Harvey, 2020). This method may begin to rival standard ESI-MS (Jin et al., 2019) and can also be combined with cryogenic infrared spectroscopy (Mucha et al., 2019) or with MALDI imaging (Heijs et al., 2020). In terms of glycomics of lower eukaryotes, there appear to be few studies involving IM-MS; however, sulfated N-glycans from a lepidopteran protease and oligo- and paucimannosidic N-glycans of Semliki forest virus derived from the C6/36 mosquito cell line have been detected and fragmented using this methodology (Crispin et al., 2014, 2015). The application of IM-MS to non-mammalian glycans is certainly worthy of further development, especially where there are complex mixtures of low-abundance structures.

## 13 Personal Note

It is nearly 30 years since one of us (Iain B. H. Wilson) first met David Harvey. At that time he was still in the old Department of Pharmacology in Oxford, just before his move to the Glycobiology Institute—following another former colleague from Pharmacology, the late David Wing. The move was not just physical, but also a scientific one—as he no longer analyzed lipids and cannabinoids, but began his pioneering work on mass spectrometry of glycans, heralded by a mini-review article in *Glycoconjugate Journal* (Harvey, 1992). Some years after I finished my thesis, which contained relevant data based on old-fashioned glycosidase sequencing, David Harvey was kind enough to include me as a coauthor on his paper on mass spectrometric analyses of ovalbumin glycans (Harvey et al., 2000). As mentioned above, he has led the way in using negative-mode MS of glycans, but over the past decade has been a key player in establishing IM-MS of glycans (Harvey et al., 2011); furthermore, a lasting contribution to the field is his series of reviews on MALDI-TOF MS, including biannual updates on the topic (Harvey, 2018), which meant reading and summarizing hundreds of papers—a feat which helps to drive forward glycomics as a field.

## 14 Conclusion

In this brief survey focusing on the use of negative-mode MALDI-TOF MS for invertebrate, fungal, and protist N-glycomics, we hope to convince the readers that this approach—although not so often used—is incredibly valuable for defining N-glycan structures in a wide variety of species. Other negative-mode mass spectrometric approaches (ESI, IM, and FT) yield also important insights into lower eukaryotic glycomes. The resulting sum of data shows the complexity of nonvertebrate N-glycomes and continues to demonstrate that Nature keeps on reshuffling the set of basic monosaccharide and nonsaccharide building blocks resulting in a huge variety of cell-, protein-, stage- and species-specific glycoforms whose functions are only beginning to be appreciated.

## Figures and Tables

**Table 1:** Examples of negative-mode off-line MALDI-TOF MS and on-line ESI-MS of nonvertebrate N-glycans

Species	Phylum	N-glycan type	Modifications	Reference
*Trichomonas vaginalis* (protist parasite) MALDI-TOF-MS	Metamonada	Paucimannosidic, Pseudohybrid	PE, PO_4_	Paschinger et al. (2012)
*Dictyostelium discoideum* (social amoeba) MALDI-TOF-MS Figure 1	Amoebozoa	Oligomannosidic	PMe, SO_4_, GlcNAc-1-P, Bisecting GlcNAc	Hykollari et al. (2013, 2014) Hykollari, Paschinger, et al. (2017)
*Penicillium verrucosum* (filamentous fungus) MALDI-TOF-MS, ESI-MS Figure 8	Ascomycota	Oligomannosidic	PE, Bisecting Gal*f*	Hykollari et al. (2016)
*Caenorhabditis elegans* (model organism) ESI-MS Figure 8	Nematoda	Paucimannosidic	Bisecting Gal, αGal, Core GalαFuc	Yan, Brecker, et al. (2015); Yan, Jin, et al. (2015), Yan, Vanbeselaere, et al. (2018); Yan, Wang, et al. (2018)
*Dirofilaria immitis* (canine heartworm) MALDI-TOF-MS, ESI-MS Figure 5	Nematoda	Anionic	GlcA	Martini et al. (2019)
*Trichuris suis* (porcine whipworm) MALDI-TOF-MS Figure 2	Nematoda	Paucimannosidic	Core αFuc	Wilson & Paschinger (2016)
*Crassostrea virginica* (Eastern oyster) MALDI-TOF-MS Figures 3 and 4	Mollusca	Anionic	SO_4_, Blood group A, MeGalNAc	Kurz et al. (2013)
*Volvarina rubella* (Marine snail) MALDI-TOF-MS, ESI-MS, Figures 3, 5, 7 and 8	Mollusca	Neutral, Anionic, Zwitterionic	GlcA, MeAEP, Core βMan	Eckmair et al. (2016)
*Aedes aegypti* (mosquito) MALDI-TOF-MS, NSI-MS Figure 2	Arthropoda	Anionic	GlcA, SO_4_	Kurz et al. (2015)
*Apis mellifera* (honeybee) MALDI-TOF-MS, ESI-MS Figures 5 and 8	Arthropoda	Neutral, Anionic, Zwitterionic	GlcA, PE, SO_4_, Core βMan	Hykollari et al. (2018, 2019)
*Lymantria dispar* (gypsy moth) MALDI-TOF-MS Figure 5	Arthropoda	Neutral, Anionic, Zwitterionic	GlcA, PC, SO_4_	Stanton et al. (2017)
*Holuthuria atra* (sea cucumber)	Echinodermata	Anionic	NeuGc, SO_4_, Lewis A	Vanbeselaere et al. (2020)
MALDI-TOF-MS, ESI-MS Figure 4 *Ophiactis savignyi* (brittle star) MALDI-TOF-MS, ESI-MS Figures 3, 4, 6, and 8	Echinodermata	Anionic	MeNeuGc, NeuGc, PO_4_, SO_4_	Eckmair et al. (2020)

*Note*: A summary of different N-glycan types and relevant investigated modifications in different eukaryotic species analyzed in our studies by off-line MALDI-TOF MS, NSI-MS, and/or on-line ESI-MS; for data examples, see Figures 1–8. The list of N-glycan types and modifications listed excludes those identified solely by positive-mode MS.

Abbreviations: ESI-MS, electrospray ionization mass spectrometry; MALDI-TOF MS, matrix-assisted laser desorption/ionization time-of-flight mass spectrometry; NSI-MS, nanospray ionization mass spectrometry.

## Acronyms

ATT: 6-aza-2-thiothymine
ESI-MS: electrospray ionization mass spectrometry
FT-ICR: Fourier-transform ion cyclotron resonance
GC-MS: gas chromatography
HIAX: hydrophilic interaction/anion exchange
HPLC: high-performance liquid chromatography
MALDI-TOF MS: matrix-assisted laser desorption/ionization time-of-flight mass spectrometry
MeAEP: methylaminoethylphosphonate
NMR: nuclear magnetic resonance
NSI-MS: nanospray ionization mass spectrometry
PC: phosphorylcholine
PE: phosphoethanolamine
RP: reversed phase
